# The Chemo-Gut Probiotic Trial Protocol: A Double-Blind Randomized Controlled Trial Investigating the Effects of a Multi-Strain Probiotic on Gut Microbiota, Gastrointestinal Symptoms, and Psychosocial Health Among Cancer Survivors

**DOI:** 10.3390/mps9050152

**Published:** 2026-10-20

**Authors:** Julie M. Deleemans, Raef Mina, Athina Spiropoulos, Oluwaseyi A. Lawal, Raylene A. Reimer, Safiya Karim, Bill Richardson, Linda E. Carlson

**Affiliations:** 1 Department of Oncology, Cumming School of Medicine, University of Calgary, Calgary, AB T2N 4N1, Canada; julie.deleemans@ucalgary.ca (J.M.D.); raef.mina@ucalgary.ca (R.M.); oalawal@ucalgary.ca (O.A.L.); safiya.karim@cancercarealberta.ca (S.K.); 2 Department of Psychology, University of Calgary, Calgary, AB T2N 1N4, Canada; athina.spiropoulo1@ucalgary.ca; 3 Department of Biochemistry & Molecular Biology, Cumming School of Medicine, University of Calgary, Calgary, AB T2N 2T8, Canada; reimer@ucalgary.ca; 4 Arthur J.E. Child Comprehensive Cancer Centre, Cancer Care Alberta, Alberta Health Services, Calgary, AB T2N 5G2, Canada; 5 Patient and Family Advisory Network, Cancer Care Alberta, Alberta Health Services, Calgary, AB T2N 5G2, Canada; willard.richardson@gmail.com

**Keywords:** probiotics, cancer survivorship, gastrointestinal, psychosocial, gut microbiome, symptom management, integrative oncology

## Abstract

**Background**: Survivors of cancer experience chronic gastrointestinal (GI) and psychosocial health symptoms and reduced gut microbial diversity. This may compromise their long-term health and wellbeing. No studies have investigated probiotics for managing both GI and psychosocial symptoms together, or their effects on the composition of the gut microbiota in post-treatment cancer survivors. **Aims:** To investigate the effects of a probiotic vs. placebo on (i) abdominal pain and depressive symptoms (primary outcomes); (ii) (a) GI (i.e., gas/bloating, diarrhea, and constipation), and psychosocial (i.e., anxiety, cognitive function, and fatigue) symptoms and (b) gut microbiota composition; and (iii) relationships between bacteria and GI and psychosocial symptoms. **Methods:** This double-blind, placebo-controlled, two-arm, randomized trial will recruit N = 66 participants allocated 1:1 to the probiotic or placebo group for a 12-week trial. Adult survivors diagnosed with a solid tumor or blood cancer who have completed chemotherapy within the last 5 years and show elevated GI or psychosocial symptoms will be included. The probiotic capsule contains a blend of *Lacticaseibacillus rhamnosus*, *Lactobacillus helveticus*, and *Bifidobacterium animalis* subsp. *lactis* strains, ingested orally once daily. Stool samples will be collected at baseline and week 12 and analyzed using 16S rRNA gene sequencing. GI and psychosocial surveys will be completed at baseline and at weeks 6 and 12. Descriptive statistics, frequencies, paired-samples *t*-tests, linear mixed models, and Spearman’s correlational analyses will be used. **Conclusions:** We explore a novel, microbiota-based intervention for chronic GI and psychosocial symptoms following cancer treatment. Our findings and commitment to person-centered knowledge translation via creating informational materials will enable people to make informed decisions about their health.

## 1. Introduction

In 2025, there were approximately 254,800 new cancer diagnoses in Canada, and for many cancer types, the 5-year survival rates have increased to over 85% post-treatment [1]. While cancer survival rates have improved, many cancer survivors (i.e., people with cancer who have completed treatment) live with chronic and distressing GI and/or psychosocial symptoms for months or years after cancer treatment has ended. This can adversely impact quality of life, compliance with maintenance therapies, and follow-up care, and has economic implications such as cancer-related disability and health care service usage [2]. Treatment for GI symptoms typically involves pharmacological strategies, such as laxatives or anti-diarrheal medications, although less than half of people with functional GI disorders express satisfaction with the efficacy of such treatments [3]. Treatment for psychosocial symptoms in people with cancer may include pharmacological options such as antidepressants and anxiolytics, as well as strategies such as cognitive behavioral therapy [4,5].

Many people express interest in, and preference for, complementary medicine strategies to cope with symptoms and feel more in control of managing their cancer experience [6]. A systematic review of complementary therapy (CT) use among people with cancer worldwide found that 51% of patients reported using CTs at some point, with use ranging from 16.5% to 93.4% depending on the study. Primary reasons for CT use included wanting to address complications associated with cancer and therapy (62.3%), improving perceived influence on general health (55.7%), and the desire to take control of one’s treatment (45.9%) [6]. Other studies have shown that CT users were also more likely to be experiencing more physical symptoms, anxiety, and depression, which strongly predicted greater CT use, with the most popular being natural health products such as probiotics [7]. Hence, further research into novel microbiota-based therapies for addressing GI and psychosocial symptoms is warranted.

A comprehensive review by Andreyev et al. concluded that chronic GI issues are underestimated in the literature and in clinical trials, underreported by patients, and that commonly used GI questionnaires generally fail to investigate key issues that are distressing to patients [8]. The prevalence, severity, and type of chronic GI issues vary depending on cancer type and treatment but are estimated to occur in 15–66% of survivors [8]. Our previous work was the first to characterize and specify the type, severity, and duration of these post-treatment GI symptoms. Participants, who were primarily 6 months to 3 years post-treatment and had a variety of cancer types, reported multiple symptoms, the most common being constipation (53.6%), diarrhea (50.5%), and abdominal pain and bloating (54.9%). When asked to rate the degree of interference from GI symptoms, most rated theirs as moderate (29.7%), although nearly one-quarter (22.2%) indicated high to extreme GI symptom interference severity. The mean duration of GI symptoms experienced since the end of treatment was 30.5 months (SD = 33.4), ranging from 0 (i.e., finished less than 1 month) to 144 months [9].

Importantly, research suggests a link between the gut microbiota and psychosocial and gastrointestinal (GI) symptoms in people with cancer [10,11], which represents a novel area of exploration. For example, previous literature suggests that chemotherapy-induced disturbances to the gut microbiota can have a detrimental impact on the patient’s psychosocial outcomes and cognitive functioning [12]. Our previous work found that survivors experienced more GI symptoms and reported worse mental and physical health symptoms compared to healthy population norms, and more severe GI symptoms were associated with poorer mental and physical health. Moreover, regardless of other types of treatments received, chemotherapy was associated with more severe abdominal pain, gas, and bloating, while radiation therapy was associated with greater diarrhea severity [9].

Consistent with this, our previous research investigated associations between factors related to cancer treatment (specifically chemotherapy and time off treatment), the gut microbiota, and GI and psychosocial symptoms in hematological and solid tumor cancer survivors compared to healthy controls. Survivors who were 6 months or less post-treatment had significantly lower gut microbial alpha diversity, a marker of microbial disturbance, compared to survivors who were more than 6 months post-treatment and healthy controls. Additionally, all survivors had more severe GI and psychosocial symptoms compared to healthy controls, and specific types of bacteria were associated with certain psychosocial symptoms [12]. For instance, lower Intestinibacter abundance was associated with poorer cognitive function and more depressive symptoms [12], suggesting that certain bacteria could potentially be targeted to improve these outcomes. This notion is supported by the chemotherapy-driven dysbiosis of the microbiota–gut–brain axis model, which suggests that chemotherapy results in elevated bacterial toxins in the body, such as lipopolysaccharide (LPS), by increasing intestinal permeability. These bacterial toxins enter the bloodstream, which leads to an increase in (1) pro-inflammatory cytokines and (2) dysregulation of the HPA axis, ultimately leading to systemic inflammation in the body [10,13]. Such inflammatory mechanisms ultimately trigger the stress response by increasing cortisol levels, which then leads to worsening inflammation in the body. As a result, disturbances to the gut microbiome, combined with dysregulation of the immune system and the HPA axis, ultimately lead to changes in serotonin and brain-derived neurotrophic factor (BDNF) levels, leading to psychological and cognitive problems among cancer patients [10,13,14].

This study was the first to show potential long-term disturbance (dysbiosis) of the gut microbiota following chemotherapy, and associations between specific psychosocial symptoms and bacteria in cancer survivors. Findings also suggest that the first 6 months to 1 year post-treatment may be a crucial time to implement microbiota-based interventions, such as probiotics or special diets, to potentially prevent or reverse GI and psychosocial symptoms in cancer survivors.

Hence, previous research highlights a crucial need for simultaneously addressing both GI and psychosocial symptoms in survivors of cancer, since these symptoms tend to co-occur, potentially in a bidirectional manner. Importantly, a systematic review of prebiotic and probiotic interventions for treating GI and/or psychosocial symptoms in people with cancer found that probiotic supplementation improved GI symptoms and QOL [15]. Moreover, a second systematic review revealed that the use of probiotic interventions significantly reduced chemotherapy-induced GI symptoms, such as constipation and bloating, among leukemia patients [16]. However, to date, most probiotic intervention studies focus on people in active treatment who have a diagnosis of colorectal cancer, leukemia, or other cancers of the pelvic region, with few studies involving survivors and using more rigorous randomized, controlled designs.

Several studies have looked at the efficacy of probiotic interventions in alleviating psychological distress among cancer patients. For example, a double-blind, placebo-controlled trial revealed that the tested probiotic supplement was effective at reducing psychological distress among cancer patients undergoing chemotherapy [17]. However, to date, only a few studies have examined the effects of probiotic interventions on psychosocial symptoms in cancer survivors. For example, Lee et al. [18] measured the effects of a 12-week probiotic intervention (Lacidofil^®^, consumed twice daily) or placebo on fatigue, anxiety, depression, and GI symptoms in a group of 60 colorectal cancer survivors who were within 2 years post-treatment. Following probiotic intervention, participants improved significantly on all psychosocial outcomes and reported reduced GI symptoms. While this study provided essential knowledge regarding the use of probiotics for the management of GI and psychosocial symptoms, the cohort consisted exclusively of colorectal cancer survivors, which limits the generalizability of the findings. Furthermore, based on our previous work, it is evident that survivors of other diagnoses who previously received chemotherapy (e.g., breast and blood cancers) also frequently experience GI and psychosocial symptoms following treatment. Hence, further research is needed in a more diverse cohort of cancer survivors across multiple cancer types. 

While other studies have examined the effects of probiotics on GI symptoms in people undergoing active treatment (e.g., radiotherapy), no studies have investigated the potential use of probiotics as a microbiota-mediated approach to managing GI and psychosocial symptoms in a diverse group of post-treatment cancer survivors. Our study is the first to use a placebo-controlled, randomized controlled trial (RCT) design and to explore the impact of this intervention on both GI and psychosocial symptoms, as well as the gut microbiota, using a comprehensive battery of outcomes in a cohort of cancer survivors. As such, we hypothesize that:

1. Intake of the multi-strain probiotic for 6 and 12 weeks will be associated with reduced abdominal pain and depressive symptoms among cancer survivors;
2. Intake of the multi-strain probiotic for 6 and 12 weeks will be associated with reduced gastrointestinal (gas/bloating, diarrhea, and constipation) and psychosocial (anxiety, cognitive function, and fatigue) symptoms.

## 2. Materials and Methods

This study was approved by the Health Research Ethics Board of the Alberta Cancer Committee (Ethics ID: HREBA.CC-22-0289). The trial protocol registration is available on ClinicalTrials.gov, NCT06088940 (18 October 2023). This protocol paper is presented in accordance with the Standard Protocol Items: Recommendations for Interventional Trials (SPIRIT) protocol guidelines, which are standard guidelines that outline the minimum content needed in a protocol paper [19]. This trial was funded through the Tom Baker Cancer Centre Investigator Initiated Trials program, funded by the Alberta Cancer Foundation.

### 2.1. Objectives

A summary of the study objectives and endpoints is in Table 1.

### 2.2. Study Design

**Figure 1:**
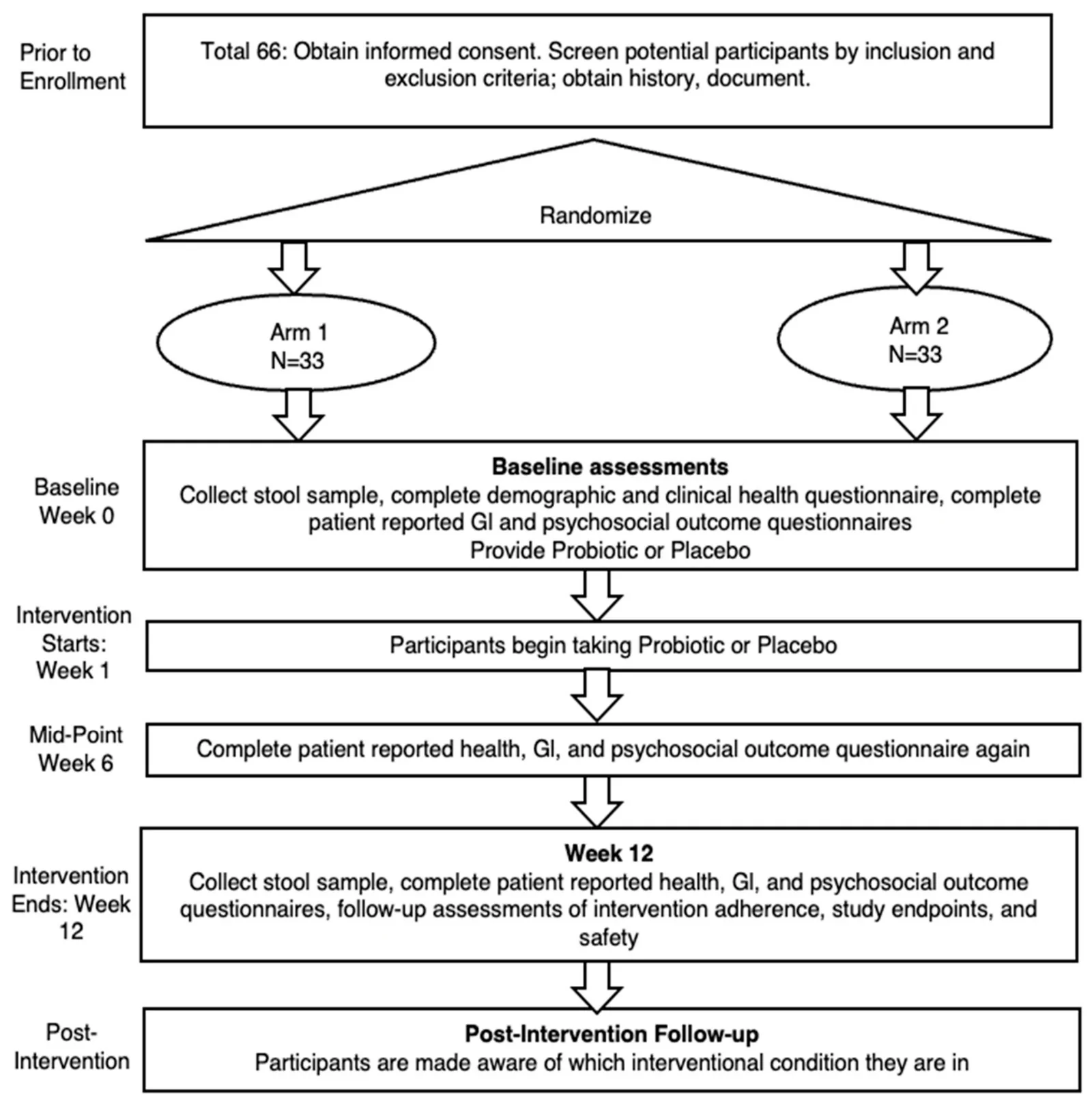
Study design.

A placebo-controlled randomized double-blind study design will be used. A flow chart illustrating the trial design is outlined in Figure 1.

### 2.3. Participants

A total of 66 participants will be randomized to receive either the probiotic or the placebo (*n* = 33 per group), administered daily for 12 weeks. Male and female participants who are 18 or older and who are within five years of their last cancer treatment will be enrolled. 

### 2.4. Inclusion Criteria

To be eligible for enrolment in this study, participants must meet all of the following criteria:

1. Male or female, any ethnicity;
2. Aged 18 years or older;
3. Diagnosed with hematological cancers (leukemia, Hodgkin’s, and non-Hodgkin lymphoma), breast, osteosarcoma, Ewing’s sarcoma, gynecological (cervical, endometrial, or uterine), prostate, testicular, esophageal, stomach, laryngeal, oral pharyngeal, or thyroid cancer;
4. Stages I–IV, including metastatic if stable and off treatment;
5. Have received and completed chemotherapy (with or without radiation, surgery, immunotherapy, or hormone therapy);
6. Within 5 years from their final cancer treatment, with emphasis placed on recruiting those within the first year post-treatment;
7. Not currently pregnant or planning to become pregnant during the 12-week study;
8. Evidence of clinically elevated levels of GI and/or psychosocial symptoms as determined using PROMIS short forms for GI symptoms, mental health, and physical health;
9. Able to provide stool samples;
10. Fluent in English and with access to a computer, smartphone, or tablet with internet access to complete questionnaires;
11. Able to provide written informed consent.

These criteria were established to recruit a diverse sample with cancer types that have higher 5-year overall survival rates and are consistent with cohorts examined in other probiotic studies and previous research. Chemotherapy is a known treatment factor impacting symptoms of interest, and treatment must be completed to avoid unknown interactions with anti-cancer therapies. Pregnant women were not included due to safety precautions and because pregnancy can impact the gut microbiota. Setting a minimum threshold for GI and/or psychosocial symptoms will ensure that symptoms are present for treatment and help prevent floor effects during analysis.

### 2.5. Exclusion Criteria

Exclusion criteria for participants include the following:

1. Diagnosis of central nervous system (CNS) tumors or colorectal cancer;
2. Currently receiving immunotherapy;
3. Having taken antibiotics and/or a daily probiotic (including probiotic yogurt) within the 1 month prior to study participation;
4. Diagnosed with irritable bowel syndrome or inflammatory bowel disease prior to being diagnosed with cancer;
5. Diagnosed with a developmental/cognitive delay prior to cancer (e.g., autism spectrum disorder, Down syndrome) or another psychological condition that, in the opinion of the qualified investigator (QI), may adversely affect the participant’s ability to complete the study or its measures or pose a significant risk to the participant;
6. Being immunodeficient and immunocompromised (e.g., HIV or long-term corticosteroid treatment);
7. Having a known or suspected food allergy or intolerance or hypersensitivity to an ingredient in the study products (milk, soya, magnesium stearate, potato starch, hypromellose, titanium dioxide, ascorbic acid, maltodextrin, sucrose, trehalose, and yeast);
8. Known type 1 or 2 diabetes, coronary artery disease, some blood/bleeding disorders, liver and kidney disorders, or any other condition that, in the opinion of the QI, may pose significant risk to the participant;
9. Currently enrolled in another clinical trial involving an intervention that may affect the study outcomes.

These criteria were selected because CNS or colorectal diagnosis and treatment (e.g., cranial radiation and colon resection) may affect cognition, mood, and GI function independently of chemotherapy. Antibiotics are associated with gut dysbiosis (disturbance), while commercial probiotics may confound the effects of the specialized formula used in this study. A prior GI disorder is exclusionary, as it is not caused by chemotherapy treatments if such GI symptoms occur prior to cancer. Patients who are immunodeficient due to conditions such as HIV are ineligible to reduce the further risk of potential immune compromise, since probiotics are contraindicated in some immune-compromised conditions.

### 2.6. Recruitment

All study procedures will be conducted remotely from the participant’s home, including screening, consent, stool sample collection, online questionnaires, and product dosing. Participants will be recruited from (i) the Arthur Child Comprehensive Cancer Centre in Calgary, Canada (i.e., via tumor-specific clinics, psychosocial oncology, the Alberta Complementary Therapy and Integrative Oncology Centre, and digital signage), (ii) via advertisements on social media and through relevant patient advocacy groups (e.g., Wellspring Calgary and Localife Calgary), and (iii) invitations sent to people who previously participated in the Chemo-Gut project (n > 300), since many had expressed interest in being involved in further research of this nature, who had given consent to be contacted. To reach potential participants through oncology clinics and digital signage (the first strategy), participants may self-refer using an online contact form. Those recruited via social media and community groups (the second strategy) may contact the study team directly. Participants who previously participated in the Chemo-Gut project (the third strategy) will be contacted via email. The study opened for enrolment in May 2024. It is estimated that it will take approximately 24 months to complete data collection.

### 2.7. Probiotic Intervention

The interventional probiotic contains a proprietary blend of *Lacticaseibacillus rhamnosus*, *Lactobacillus helveticus*, and *Bifidobacterium animalis* subsp. *lactis* strains that were selected because they have been studied extensively with various patient cohorts and are known psychobiotics [20]. The total dose per capsule is 9 × 10^9^ colony-forming units (CFUs) at the end of the shelf life. The placebo (maltodextrin, an identically formulated and packaged inactive substance) will be identical to the probiotic in shape, appearance, and texture, such that participants will not be able to discern a difference between the probiotic and the placebo. Additionally, an equivalent amount of maltodextrin was added to match the weight of the placebo product. Both the probiotic and placebo will be a size 1 capsule that will be ingested orally. The dose is guaranteed by the manufacturer through periodic product stability and quality testing throughout the duration of the study. There is currently no gold standard for probiotic intervention, and the optimal dose, bacterial strains, and duration vary depending on the patient cohort and symptoms to be treated. However, we have selected a 12-week intervention time frame based on previous studies showing efficacious results following probiotic intervention in cancer survivors over that length of time [15,18].

### 2.8. Randomization, Allocation Concealment, and Masking

Participants will be randomized individually upon enrollment, after completing baseline assessments, into the probiotic or placebo condition in a 1:1 allocation ratio using a computer-generated stratified randomization strategy to balance age and sex, which is implemented within REDCap Version 16. Packaging will be identical and labeled using six four-digit codes so as not to be identified by participants or blinded members of the study team. Both the probiotic and placebo will be stored in the plastic bottles in which the products are packaged. To maintain masking, once randomized, a research assistant with no other study involvement will be the only one to learn of the group assignment and assign the probiotic or placebo bottle of pills for each participant to their study package. The study coordinator and research assistant who will have direct contact with participants will remain masked to participants’ condition throughout, as will the participants themselves. The study coordinator will be unmasked only after all data collection is complete. The biostatistician will remain masked to the condition during data analysis, with groups only identified as group A or group B in the database (by flipping a coin to determine which is the probiotic or placebo).

### 2.9. Product Handling

The investigational product was delivered to the study site under refrigerated, controlled conditions, under the supervision of the manufacturer. The study product will be stored in a locked, temperature-monitored fridge accessible only to research staff at the Arthur Child Cancer Center. The fridge’s internal temperature is between 2–8 °C, and a TempTale temperature sensor will be placed inside the refrigerator to continuously monitor the internal temperature. Participants are instructed to store the study product at ambient room temperature during the intervention period (20–25 °C).

### 2.10. Study Procedures

Once a participant has been enrolled in the study, a date and time will be scheduled for their study kit to be delivered and for baseline questionnaires to be administered via REDCap. The study kit will contain a stool sample collection kit and the probiotic or placebo in a 12-week supply. Participants will be instructed to take 1 capsule orally each day with a large glass of water in the morning, either on an empty stomach or during mealtime (i.e., first meal of the day) for 12 weeks. If participants notice stomach issues when taking the study product on an empty stomach, they will be instructed to take it with food. Participants will also be instructed to store the study product in the fridge.

All participants will receive the standard dose as indicated by the manufacturer for the duration of the study period. Both the probiotic and placebo will be manufactured and supplied by Lallemand Health Solutions Incorporated [21]. A participant is considered to have completed the study if they have completed all study activities, including the last questionnaire and sample collection, as shown in Table 2. At the end of the study, participants will be asked to return their study product supply with any unused capsules. Any unused product will be returned to the investigators to measure compliance with the intervention. Participants with >20% of capsules remaining will be considered non-compliant with the intervention and excluded from subsequent analysis.

### 2.11. Study Assessments

#### 2.11.1. Demographics and Clinical Data

Clinical and demographic factors, including current age, age at diagnosis, income, occupation, ethnicity, education, cancer diagnosis and treatments received, history of treatment-related mucositis, current diet and exercise behaviors, height and weight (to calculate the body mass index), antibiotic and probiotic use within the last 2 years, tobacco use, birth delivery mode (i.e., caesarean section vs. vaginal), and breastfeeding during infancy, will be collected, as these variables are known to impact the gut microbiome. 

The use of all medications, including prescription medications, over-the-counter drugs, and natural health products consumed over the course of the trial, will be monitored in a health questionnaire administered at 3 time points during the study (baseline, week 6, and week 12). The survey will include questions related to current medication use and other treatments received from health care providers that pertain to the management of GI and/or psychosocial symptoms (e.g., antidepressants, laxatives, anti-diarrheal medications, pro-motility agents, etc.).

#### 2.11.2. Psychosocial and GI Outcomes

Psychosocial and GI health outcomes will be measured via the NIH Patient-Reported Outcomes Measurement Information System (PROMIS). PROMIS is a set of person-centered measures that evaluates physical, mental, and social health, and it is a valid measure of psychosocial and physical health outcomes among adult cancer patients and patients with GI disorders such as irritable bowel syndrome and Crohn’s disease [22,23,24]. These specific outcome measures have been selected as previous research suggests that gut microbiota disturbance is associated with symptoms of anxiety and depression [25], fatigue [26], cognitive function [14], and GI disturbance [27,28].

### 2.12. Primary Outcomes

Depression will be measured using the PROMIS—Ca Item Bank v1.0—Emotional Distress—Depression questionnaire, which contains 30 items to assess the presence and severity of depression. Statements are rated on a 5-point scale from “never” (1) to “always” (5). 

Belly Pain—Scale v1.0, 5a: The PROMIS Belly Pain scale is a validated measure and has been used to evaluate GI symptoms in patients with irritable bowel disease and Crohn’s disease [23,24]. We will use this measure to assess the frequency, severity, and degree of interference for belly pain in cancer survivors. This scale includes 5 statements relevant to belly pain, which are rated on a 5-point scale from “not at all” (1) to “very much” (5). 

### 2.13. Secondary Outcomes

Gas and Bloating—Scale v1.1, 13a: The PROMIS Gastrointestinal Gas and Bloating scale is a validated measure and has been used to evaluate GI symptoms [23,24]. We will use this measure to assess the frequency, severity, and degree of interference from gas and bloating symptoms in cancer survivors. This scale includes 13 statements relevant to gastrointestinal gas and bloating, which are rated on a 5-point scale from “not at all” (1) to “very much” (5). 

Constipation—Scale v1.0, 9a: The PROMIS Gastrointestinal Constipation scale is a validated measure and has been used to evaluate GI symptoms [23,24]. We will use this measure to assess the frequency, severity, and degree of interference from constipation symptoms in cancer survivors. This scale includes 9 statements relevant to constipation, which are rated on a 5-point scale from “never” or “not at all” (1) to “always” or “very much” (5).

Diarrhea—Scale v1.0, 6a: The PROMIS Gastrointestinal Diarrhea scale is a validated measure and has been used to evaluate GI symptoms [23,24]. We will use this measure to assess the frequency, severity, and degree of interference from diarrhea symptoms in cancer survivors. This scale includes 6 statements relevant to diarrhea, which are rated on a 5-point scale from “not at all” (1) to “very much” (5). 

Anxiety will be measured using the PROMIS v1.0—Anxiety—Short Form 8a questionnaire, which contains 8 items that relate to symptoms of anxiety. Statements are rated on a 5-point scale from “never” (1) to “always” (5).

Fatigue will be measured using the PROMIS: Item Bank v1.0—Fatigue—Short Form 8a scale, which includes 8 statements related to fatigue frequency and intensity. Statements are rated on a 5-point scale from “not at all or never” (1) to “very much or always” (5).

Cognitive function will be measured using the PROMIS: Item Bank v2.0—Cognitive Function, which includes 32 statements related to various areas of cognitive function, including the frequency and severity of cognitive concerns. Items are rated on a 5-point scale ranging from “very often (several times a day)” (1) to “never” (5).

### 2.14. Exploratory Outcomes

#### Gut Microbiota Profiling

Participants will be instructed to use the investigator-provided stool collection kit to collect stool samples at home. The sample is placed in a sterile conical tube, placed in a biohazard bag, and stored in the participant’s freezer until the date and time of the predetermined pick-up from their home (not more than 3 days from the time of collection). Samples are picked up by research personnel and immediately transported on ice to the University of Calgary Faculty of Kinesiology and stored at −80 °C until analysis. Gut microbial profiling will be conducted at Lallemand Health Solutions (Montreal, QC, Canada) using established methods [29,30]. Bacterial genomic DNA (gDNA) will be extracted from fecal matter, quantified using the PicoGreen DNA quantification kit (Invitrogen, Carlsbad, CA, USA), and stored at −20 °C. The bacterial composition will be assessed by sequencing the V3 and V4 regions of the 16S rRNA gene using the MiSeq System (Illumina, San Diego, CA, USA). The probiotic strains will be detected and quantified in the fecal samples using the CFX384 Real-Time PCR System and strain-specific primers.

### 2.15. Study Procedure Timeline

The schedule of enrolment, interventions, and assessments utilized to address primary and secondary outcomes is presented in Table 2.

### 2.16. Sample Size Determination

Using G*Power 3 version 3.1.9.7 [31], we calculated that a minimum sample size of N = 54 is needed. Using a linear mixed model (LMM) within-between subjects’ interaction a priori analysis, with alpha set at 0.05 and a power of 0.90, a minimum sample size of N = 54 is needed to detect a medium between-groups effect size (Cohen’s f = 0.25). We will aim to recruit a total of N = 66 survivors for the RCT, with n = 33 participants allocated to the treatment group and n = 33 to the control group in a 1:1 treatment allocation, to allow for 20% attrition. This sample size was calculated based on the co-primary outcomes of abdominal pain and depressive symptoms improving with a medium effect size following probiotic intervention and is consistent with previous similar studies [18]. Notably, as we are using methods previously established in the Chemo-Gut pilot study [12] that showed very high (>90%) retention, we are confident that similar rates of retention will be achieved using the same approach for this intervention study.

### 2.17. Data Analysis

An interim analysis may be conducted periodically during the study to evaluate demographic and clinical characteristics, intervention adherence, and the baseline characteristics of participants. Results may be submitted for conference presentations. However, no unmasking will occur; therefore, between-group comparisons will not be conducted.

When data collection is complete, an intent-to-treat (ITT) analysis will be conducted, including all participants with baseline data. Missing data will be handled using multiple imputation (MI) with chained equations (m = 20; predictive mean-matching, donor pool = 5), which is valid under a missing-at-random (MAR) assumption. Then, a per-protocol analysis will be conducted, such that only participants who consumed at least 80% of the probiotic or placebo will be included in the full analysis. Tests of normality will be used to explore the data, and where necessary, non-parametric tests will be used. Descriptive statistics will be presented as percentages, means, and standard deviations. For linear mixed model (LMM) analysis and paired-sample *t*-tests evaluating primary and secondary outcomes, statistical significance will be set at alpha = 0.05 using two-tailed tests, with a 95% confidence interval. This approach is adopted as the standard to maintain conservatism and allow for the detection of potential effects in either direction. 

*Primary Endpoint: The effects of probiotics on abdominal pain and depressive symptoms* will be measured using the Patient-Reported Outcomes Measurement Information System (PROMIS) questionnaires [32]. PROMIS measures produce a numerical (continuous) score with a mean value of 50. Scores greater than 50 represent more severe symptomology. PROMIS scores will be calculated at baseline, week 6, and week 12. Outliers ≥3 standard deviations from the mean will be removed from subsequent analysis for that particular variable. Descriptive statistics and mixed-effects methods with a random intercept model, which accounts for the variances between participants and within participants, will be used. Each PROMIS outcome (abdominal pain, depressive symptoms, anxiety, cognitive function, fatigue, gas/bloating, constipation, and diarrhea) will be analyzed with a linear mixed-effects model (LMM) using restricted maximum likelihood: *fixed effects*: intervention group (probiotic vs. placebo), time (baseline, 6 weeks, 12 weeks), group × time interaction, and baseline score (centered). *Random effects*: participant-level intercept; a random time slope will be added if it improves model fit by ≥2 AIC units. We will follow up with a pair-wise contrast of the group’s estimated marginal means (EMMs), applying the Kenward–Roger degrees-of-freedom correction and a Bonferroni adjustment where applicable. Results will be reported as estimated marginal means ± SE, F-statistics, two-tailed *p*-values (α = 0.05), and 95% confidence intervals. Effect sizes will be reported using Cohen’s f (i.e., 0.10 = small, 0.25 = medium, and 0.40 = large effect sizes). Potential covariates were specified a priori based on the substantive literature indicating potential confounding: age (years), sex (male/female), and months since treatment. No data-driven selection will be undertaken. Exploratory interaction terms (e.g., group × sex) will be added one at a time; significant interactions will be probed via simple-effects decomposition. Per PROMIS recommendations, a change in the mean score of at least 3 points from baseline is considered a clinically meaningful difference in change scores [32].

*Secondary Endpoint B. The effects of probiotic intervention on gut microbiota* will be evaluated using 16S rRNA V3–V4 amplification gene sequencing to analyze gut microbiota composition and to examine global changes in samples between groups and across time. Additionally, 16S rRNA sequencing is a very commonly used analytical strategy and will allow our results to be easily compared to the other published literature. Stool samples for gut microbiota analyses will be collected at baseline and week 12 (post-intervention) using methods reported in our previous studies [12]. Bioinformatics analyses will be conducted as described in our previous work [12]. Descriptive statistics and *t*-tests (independent and paired-sample) will be used to investigate between- and within-group differences and to calculate intervention effect sizes.

*Subgroup analyses* may be conducted based on participant age to stratify middle-aged and older adults, since the gut microbiota of older adults has been shown to differ from that of younger and middle-aged adults [33]. Subgroup analyses may also be conducted based on sex (male versus female) to investigate differences in symptomology and gut microbiota composition. Time off treatment (i.e., the first year post-treatment versus >1 year post-treatment) may be explored based on findings from our previous work suggesting differences in gut microbiota composition within these post-treatment time frames [12]. Primary endpoint analyses will be conducted using the IBM SPSS v.29 Statistical Software Package. Secondary endpoint analyses will be conducted using validated, dedicated bioinformatics and R statistical software (version R-4.6.1), as well as established analytical pipelines.

### 2.18. Risk–Benefit Analysis

As this study involves a minimally invasive dietary supplement, the potential risks (i.e., gas, bloating, and upset stomach) are minimal but will be mentioned in the consent form and tracked throughout the study. Participants are asked to inform the study team of any adverse events via email. 

Benefits of study participation may include reduced GI symptoms and improved psychosocial health. Additionally, each participant will receive a CAD 25 gift card, a personalized report of their results, which will be interpreted for all GI, psychosocial, and gut microbiota outcomes, and helpful resources for further education, consistent with strategies used in our previous research [12].

### 2.19. Safety Reporting

Adverse Events (AEs) are undesirable symptoms that the participant experiences during a clinical trial, whether or not they are related to the investigational products. All AEs for the duration of this study will be documented in an Excel master file that contains the details of each enrolled participant. The study team will follow up with any AEs until the symptoms have resolved or returned to baseline. All events that are unexpected, potentially related to the intervention, and are considered serious will be reported to ethics and Health Canada.

The use of probiotics has been shown to be safe in various disease populations, including in people with cancer [34]. Few AEs are reported following probiotic use, and these are generally mild (e.g., upset stomach and increased flatulence); therefore, no serious AEs are expected. Participants will be asked to document adverse symptoms and inform a member of the study team via email. Distress related to post-treatment symptoms and adjusting to life after cancer is common among people with cancer. Hence, precautions are in place to ensure that if a participant is experiencing distress during the study, they can seek assistance via referral to psychosocial support services at the Arthur Child Comprehensive Cancer Centre to receive support from a dedicated clinical psychologist or social worker. Participants are asked to contact a member of the team if they require psychosocial support.

### 2.20. Participant Withdrawal and/or Discontinuation from the Study

Participants are free to withdraw from participation without penalty and without having to provide a reason at any time upon request. An investigator may discontinue or withdraw a participant from the study due to the withdrawal of informed consent (participant withdrawal for any reason), a clinical adverse event, or another medical condition such that continued participation in this study would not be in the best interest of the participant. The reason for participant discontinuation will be recorded in the study file. Participants who sign the informed consent form and are randomized but do not receive the study intervention, or those who sign the consent form, are randomized, and receive the study intervention but subsequently withdraw from the study, will be replaced. The data from participants who withdraw or discontinue from the study will be used in the analysis unless the participant requests otherwise. Adverse events necessitating discontinuation from the study are not anticipated, since probiotic interventions are generally reported to be safe in a wide range of patient cohorts, including those with cancer [34]. Participation will be discontinued if participants have a cancer relapse and need to be placed on active treatment (e.g., chemotherapy) during the 12-week intervention. Should unmasking be necessary in case of an emergency, the team member responsible for randomizing participants (i.e., the unmasked team member) will reach out to the participant and/or their health care provider directly.

### 2.21. Data Collection and Management

Once identified, potential participants will be contacted by telephone to further verify inclusion and exclusion criteria and will have the opportunity to ask questions about the study. If a participant is eligible, written informed consent will be obtained via an online form administered through Research Electronic Data Capture (REDCap™—Research Electronic Data Capture), a secure, web-based software platform developed by Vanderbilt University.

### 2.22. Data Confidentiality

The REDCap 14.5 instance used for this study is hosted and administered by the Centre for Health Informatics (CHI) at the Cumming School of Medicine, University of Calgary, Calgary, AB, Canada. REDCap is a secure, browser-based application designed to support electronic data capture (EDC) for clinical research studies, including Health Information ACT (HIA)-, Health Canada (HC)-, and Good Clinical Practice (GCP)-compliant environments. The system is approved to collect and store Level 4 data, uses two-factor authentication, and is stored and maintained on secure servers at the University of Calgary [35,36].

### 2.23. Data Monitoring

Research staff will continuously monitor participants’ data on REDCap to ensure compliance with the study protocol timeline. Participants will receive an email reminder from the research staff to ensure that participants complete the questionnaire on time. 

## 3. Results

### Trial Status

This trial began recruitment in May 2024. Due to the move to the new cancer center (i.e., The Arthur Child Comprehensive Cancer Centre, Calgary, AB, Canada), which opened in October 2024, recruitment delays were experienced. As of February 2026, thirty participants have completed the trial, and nine are currently actively enrolled. Few adverse events have been reported, such as feeling bloated or nauseous after starting the supplement. Adherence to intervention protocols has been 80–100%. The most common reasons for ineligibility in the trial include failure to meet the minimum threshold for GI and/or psychosocial symptoms, a cancer diagnosis that is not eligible (e.g., colorectal cancer), or not yet having finished active cancer therapies (e.g., chemotherapy and radiation). Enrolled participants report overall satisfaction with their experience in the study. 

We originally calculated a minimum sample size of N = 54 to detect a medium effect size for the primary objective. However, due to recruitment challenges, we have adjusted our calculations so that we now aim to recruit a total of N = 50 survivors for the RCT, with n = 25 participants allocated to the treatment group and n = 25 to the control group in a 1:1 treatment allocation. This sample size was calculated based on the co-primary outcomes of abdominal pain and depressive symptoms improving with a medium effect size following probiotic treatment. However, with the reduced sample size, the power is now expected to be reduced to the low-to-mid value of 0.80 for this study design. We will also use a linear mixed model (LMM) for the within-between-subjects interaction a priori analysis and a two-tailed hypothesis set at α = 0.05, with 95% CIs, for the final analysis to detect small to medium between-group effect sizes (Cohen’s f = 0.10 to 0.25). Despite these changes, our sample size remains consistent with previous similar studies [14]. Notably, we are using methods previously established in the Chemo-Gut pilot study [12] that showed very high (>90%) retention. While retention rates have been >90%, recruitment has been difficult primarily due to many patients remaining on immunotherapy for long periods of time after their initial treatments (e.g., chemotherapy) are complete. For safety precautions, we cannot include patients on active cancer treatments such as immunotherapy. Hence, we have been able to recruit fewer participants than expected.

## 4. Discussion

### 4.1. Implications and Dissemination

This study is uniquely innovative in exploring the impact of this intervention on both GI and psychosocial symptoms, as well as the gut microbiota, which has never been done previously using a comprehensive battery of outcomes. This study will thus be the first to determine whether probiotic intervention can be used to simultaneously reduce GI and psychosocial symptoms and enhance gut microbiota health in survivors of cancer. Our results have implications for improving symptom management and treatment adherence in clinical practice and addressing key unmet needs among cancer survivors. Notably, two of the collaborators are cancer survivors who regularly engage in patient advocacy initiatives, liaising with patient partners to ensure that patient priorities are met and that research findings are accessible to the people who need them most: patients. In particular, it was the personal experience with cancer and conversations with other survivors of the first author that highlighted the extent of the problems lingering GI issues can cause and drove the progression of this program of research, in which the current project is the next step.

The results of this study will be disseminated at national and international conferences and published in open-access, peer-reviewed journals. Additionally, this research has the potential to benefit people with cancer not only in Alberta, but throughout Canada and elsewhere. Readily available non-prescription probiotics may present a more accessible and effective alternative to prescription and other medications for symptom management. Probiotic supplements may also enhance conventional cancer treatment adherence by making treatment-related symptoms more tolerable. Furthermore, the knowledge gained from this study will help clinicians to better advise patients on the use of probiotics for symptom management and allow patients to make informed decisions about their health. Finally, consistent with our commitment to patient-oriented research, we will create patient educational materials based on the results of this study, including infographics and webinars presented to patient support groups. This ensures our findings will be accessible to patients and their loved ones, empowering them to make informed decisions about their health.

### 4.2. Limitations

While this study investigates a unique research question, it is important to note some limitations. First, given the diverse characteristics of our sample, including their cancer type, symptoms, and dietary intake, the sample is considered heterogeneous. While a heterogeneous sample is more reflective of real-world clinical settings, it may also introduce substantial clinical and statistical variability. Additionally, our primary and secondary variables are primarily self-reported, which introduces the potential for same-source bias and, as such, may potentially inflate associations between variables. Furthermore, given our reduced sample size, our statistical power is decreased, which further limits our ability to detect whether probiotics may alleviate the symptoms of interest.

## 5. Conclusions

This innovative study aims to determine whether a 12-week multi-strain probiotic (vs. placebo) intervention can be used to improve GI symptoms (i.e., abdominal pain, gas/bloating, diarrhea, and constipation) and psychosocial health (i.e., depression, anxiety, cognitive function, and fatigue) in post-treatment cancer survivors. This trial will also be the first to examine the composition of the gut microbiome within this context, to determine whether probiotics impact gut microbial composition, and whether these changes are related to GI and psychosocial symptoms. If successful, this intervention may offer a low-risk, more accessible solution to address chronic GI and psychosocial issues in cancer survivors and empower patients to make informed decisions about their health and wellbeing.

## Figures and Tables

**Table 1:** Study objectives and endpoints.

Objectives	Endpoints	Endpoint Justifications
Primary		
To investigate the effects of a multi-strain probiotic compared to placebo on abdominal pain and depressive symptoms in adult cancer survivors.	Effects of probiotics on abdominal pain and depressive symptoms at 6 and 12 weeks. Clinically meaningful effects will be defined as reductions of 3 points or more from baseline scores of abdominal pain and depressive symptoms on PROMIS measures (see Section 2.17).	These endpoints were selected as primary outcomes based on previous work in the Chemo-Gut project showing high symptom burden for both abdominal pain and depressive symptoms in survivors of cancer.
Secondary		
To investigate the effects of a multi-strain probiotic vs. placebo on (a) other GI symptoms (i.e., gas/bloating, diarrhea, and constipation) and psychosocial outcomes (i.e., anxiety, cognitive function, and fatigue); and (b) gut microbiota composition.	Clinically meaningful effects will be defined as reductions of 3 points or more from baseline score on PROMIS measures, as recommended; (b) probiotic effects on gut microbiota composition will be determined by statistically significant differences in metrics of alpha and beta diversity, differential taxonomic abundance between groups at week 12, and long-term effects from baseline to 12 weeks post-intervention.	These endpoints were selected as secondary outcomes based on findings from our earlier work that suggested a higher symptom burden for these GI and psychosocial outcomes. Gut microbiota outcomes were selected based on our previous work suggesting that the post-treatment months may be a crucial time to intervene using microbiota-based therapies such as probiotics.
Exploratory		
Explore relationships between bacterial taxa and GI and psychosocial symptoms.	Relationships between bacterial taxa and GI and psychosocial symptoms will be determined by examining statistically significant correlations between variables.	This endpoint was selected for the exploratory outcome based on our previous work suggesting relationships between specific types of bacteria and symptoms, such as depression and cognitive function.

**Table 2:** Schedule of enrollment, interventions, and assessments.

Measurement Time Points		Enrollment		Intervention Administration		
*Construct*	*Measure*	*-t*_1_ * to t*_0_	*t*_0_	*Baseline* *(t*_1_ *)*	*Week 6 (t*_2_ *)*	*Week 12 (t*_3_ *)*
Eligibility Screen	PROMIS Belly Pain (scale v1.0, 5a)	X				
Informed Consent	ICF completed online	X				
Randomization	Conducted on REDCap after enrollment		X			
***Demographic/baseline Measures***						
GI symptoms	PROMIS Belly pain (scale v1.0, 5a)			X		
Demographics and Medical History	Age, gender, marital status, education, other medical conditions, medications, height, weight			X		
Disease and diagnostic details	Type and stage of cancer, date of diagnosis, time since last cancer treatment(s)			X		
Contamination	Use of probiotics, antibiotics, prescription medications, OTC drugs, natural health products, other treatments from health care providers for symptom management			X	X	X
Pregnancy	Urinary pregnancy test			X		
Adverse events record	Collection of adverse events during the whole study				X	X
***Primary Outcomes—Self-Reported***						
Depressive symptoms	PROMIS Depression (Ca Item Bank v1.0—Emotional Distress—Depression)			X	X	X
Abdominal pain	PROMIS Belly Pain (scale v1.0, 5a)			X	X	X
***Secondary Outcomes—Self-Reported***						
Anxiety	PROMIS Anxiety (v1.0—Short Form 8a)			X	X	X
Cognitive function	PROMIS Cognitive Function (v2.0)			X	X	X
Fatigue	PROMIS Fatigue (v1.0, 8a)			X	X	X
Gas/Bloating	PROMIS Gas and Bloating (scale v1.1, 13a)			X	X	X
Constipation	PROMIS Constipation (scale v1.0, 9a)			X	X	X
Diarrhea	PROMIS Diarrhea (scale v1.0, 6a)			X	X	X
***Objective Outcome Measures***						
Gut microbiota composition	16S rRNA sequencing			X		X
Intervention compliance	≥80% of the probiotic or placebo consumed at the end of the study					X
Study metrics	Accrual, retention, data completeness, side effects					X

## Data Availability

Following completion of data collection, analyses, and publication, de-identified data will be available upon request to the corresponding author.
